# Intravitreal Anti Vascular Endothelial Growth Factor Agents in The Management of Retinal Diseases: An Audit

**DOI:** 10.2174/1874364101711010315

**Published:** 2017-10-31

**Authors:** Bassey Fiebai, Victor Odogu

**Affiliations:** 1 Department of Ophthalmology, University of Port Harcourt Teaching Hospital, , Nigeria; 2 Department of Ophthalmology, Niger Delta University Hospital, Yenagoa, Nigeria

**Keywords:** Anti vascular endothelial growth factor, Intravitreal injections, Bevacizumab, Ranibizumab, Retinal diseases, VEGF

## Abstract

**Purpose:**

The study aimed to describe our initial experience with the use of anti vascular endothelial growth factors (anti-VEGFs) in the treatment of retinal diseases.

**Methods:**

The case records of all patients who had received at least 3 doses of intravitreal anti- VEGF injections between January 2012 to December 2016 were reviewed. Information culled from the data was age, sex, indications for treatment, type of injection, presenting visual acuity, post injection visual acuity, systemic and ocular co morbidities. Results were analyzed using Statistical Package for Social Sciences (SPSS) 20.0 for Windows statistical software

**Results:**

A total of 190 injections were given during the study period, to 58 eyes of 50 patients. Twenty-eight females (56.00%) and twenty-two males (44.00%) were seen with a mean age of 59.6± 11.66. Bevacizumab was the most frequently administered anti- VEGF, 142 (74.74%) while only 48(25.26%) injections of Ranibizumab were given. Three eyes had both bevacizumab and ranibizumab (1.58%). Retinal vein occlusion 61(32.11%) was the commonest indication for the injections followed by diabetic macular edema 43(22.63%) and proliferative diabetic retinopathy 42(22.11%). Others were neovascular age related macular degeneration, neovascular glaucoma, vitreous hemorrhage, myopic choroidal neovascularization and cystoid macular edema. There was an association between age and disease, (p = 0.001). There was an improvement in visual acuity after intervention in cases with retinal vein occlusion and diabetic macular edema, and this was statistically significant. Hypertension was the commonest systemic disorder in this series 81(42.36%) and the supero-temporal quadrant 131(68.95%) was the most preferred position to administer the injection. Floaters was the commonest complication seen.

**Conclusion:**

Anti VEGFs have become an invaluable tool in the management of a number of retinal diseases in our center. However, the cost implications are a hindrance to an increased uptake of this form of treatment. Cheaper alternative preparations should be made available to encourage the uptake. Government in developing countries should be encouraged to bear the health burden of the old aged pensioner (OAP).

## 1 INTRODUCTION

Vascular endothelial growth factors (VEGF) are a key contributor in the process of angiogenesis as they promotes proliferation and vascular endothelial cell proliferation [1]. They increase vascular permeability and vasodilation required in physiological processes like lesion healing, and have also been found to play a major part in mediating active intraocular neovascurization in patients with ischaemic [2, 3]. These conditions include retinal disorders such as retinal vein occlusion(RVO), proliferative diabetic retinopathy and diabetic macula edema(DME), wet age related macular degeneration(AMD), myopic neovascularization and retinopathy of prematurity. The complications of neovascularization include neovascular glaucoma, vitreous hemorrhage and retinal detachment which are major causes of visual impairment that arises from these conditions [3].

VEGF is a 40 kDa dimeric glycoprotein that is produced by hypoxic stimulation in different cells of the retina: vascular endothelium, retinal pigment epithelial cells, Müller cells [4]. There are seven members of the VEGF family (A-F and placental growth factor) and four isoforms that are believed to play a key role in the human eye: VEGF-121, VEGF-165 (responsible for pathological ocular neovascularization), VEGF-189 and VEGF-206 [5].

Anti-vascular endothelial growth factors (anti-VEGFs) have revolutionized the management of retinal diseases in the past decade. The first VEGF-A inhibitor, bevacizumab (Avastin), was approved by the US Food and Drug Administration (FDA) in 2004 for the first-line treatment of metastatic colorectal cancer. It is a monoclonal antibody (149kDa) that binds to all isoforms of VEGF-A and is now being used as an off label intravitreal drug for the treatment of retinal vascular disorders. The first VEGF-A inhibitors in ophthalmology, pegaptanib (Macugen) and ranibizumab (Lucentis), were approved in 2004 and 2006, respectively. Ranibizumab was formulated for intraocular usage only. It is an Fab fragment of the humanized monoclonal antibody (48kDa) and has an affinity for all VEGF isoforms. The smaller molecule compared to Bevacizumab allows better retinal penetration. Aflibercept (Eylea) is a recent anti-VEGF therapy approved in 2011. It is indicated for use in the treatment of neovascular age related macular degeneration (AMD), macular edema from retinal vein occlusion, diabetic macular edema(DME) and diabetic retinopathy in patients with DME for the treatment of neovascular AMD. These agents have shown significant gain in visual acuity and improvement in morphological outcomes [6].

Together or alone with other conventional treatment (*e.g.* Laser photocoagulation and intravitreal steroids), retina specialists now have an armamentarium of antiVegf agents in the treatment of ocular neovascular disorders. The uptake of these agents has increased in developing countries [7]. However due to the costs of these drugs and the duration of treatment, compliance in a poor resource setting as seen in the developing countries is a major challenge [7].

## 2 MATERIALS AND METHODS

Case records of patients attending the retina clinic of the University of Port Harcourt Teaching Hospital who received at least 3 doses of intravitreal injection between January 2012 to December 2016 were reviewed.

The parameters evaluated included patient’s demographic data, indications for injection, type of intravitreal injection used, ocular co morbidities, systemic risk factors and ocular complications.

Intravitreal injections were all given in the operating room under strict aseptic conditions. All patients gave their informed consent. Visual acuity and intra ocular pressure were checked before and after the injections. All the injections were given using topical anesthesia (tetracaine hydrochloride, 0.5%). 10% Povidone iodine was used for skin preparation while 5% was instilled into the conjunctival sac before and after injection in all patients. No topical antibiotics were given before and after the procedure.

Dosage of intravitreal bevacizumab given was 1.25mg in 0.05ml, while 0.5mg of 0.05ml of ranibizumab was given. Injection site varied between the superotemporal quadrant, inferotemporal and superonasal depending on the surgeon’s access.

Injections were given 4mm from the limbus for phakic patients and 3.5mm for pseudophakic patients. Patients were reviewed one day, one week and one month post injection, to assess their visual acuity and check intra ocular pressure. Injections were given every 4 weeks. All patients in this study were followed up for at least 12 months.

Information from each subject was entered into a spreadsheet using the Statistical Package for Social Sciences (SPSS) 20.0 for Windows statistical software and analyzed. Comparison of variables was carried out using appropriate statistical tests. P values of <0.05 were considered statistically significant.

## 3 RESULTS

58 eyes of 50 patients were injected. A total of 190 injections were given during this period, consisting of 142 injections of Bevacizumab (74.74%) and 48 injections of Ranibizumab (25.26%), Fig. (**1**). 3 eyes had both bevacizumab and ranibizumab (1.58%).

Ninety- nine (52.11%) females received injections, while 91 males (47.89%) with a mean age of 59.6± 11.66 years (Table **1**).

The minimum follow-up time was 12 months, while the maximum was 51. The mean follow-up time was 20.05± 8.96.

Retinal vein occlusion 61(32.11%) was the commonest indication for the injections followed by diabetic macular edema 43(22.63%) and proliferative diabetic retinopathy 42(22.11%). Others were neovascular age related macular degeneration 26(13.68%), neovascular glaucoma 9(4.74%), vitreous hemorrhage 3(1.58), myopic choroidal neovascularization (1.58) and cystoid macular edema 3(1.58). Table (**1**) also shows that the supero-temporal quadrant 131(68.95%) was the most preferred position to administer the injection. The intraocular pressure was raised in 61cases (32.11%). Floaters was the commonest complication seen. Fig. (**2**) shows an association between age and disease, (p = 0.001). Hypertension was the commonest systemic disorder in this series 81(42.36%), as seen in Table (**2**).

## 4 DISCUSSION

The mean age in this series was 59.56± 11.66 years. Systemic conditions such as diabetes and hypertension which cause retinal microvascular pathologies leading to neovascularization and edema have been shown to be common in this age group [8, 9]. A similar trend was also reported in a Nigerian tertiary private eye-care facility [10].

The female preponderance seen in this study, which was not statistically significant, may be due to the same reasons noted in the previous studies done in the same area suggesting that women have a better health seeking behavior [8].

This does not suggest that there is an increase in ocular disease in females however, just that they are more willing to present themselves to seek medical attention.

**Fig. (1):**
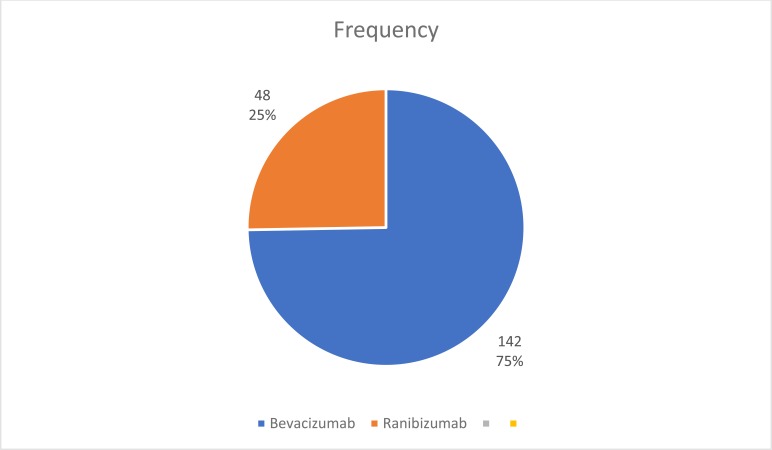
Distribution of types Of anti vegf injections.

One hundred and ninety injections were given in this series, with Bevacizumab being the most frequently injected anti Vegf (see Fig. **1**).

3 eyes of 2 patients received both injections, switching from bevacizumab to ranibizumab because of financial constraints. Similar studies reckoned that the cheaper cost of bevacizumab compared to ranibizumab explained its preference [9, 11].

Retinal vein occlusion, made up of central retinal vein occlusions, branch retinal vein occlusions and hemi retinal vein occlusions, was the commonest indication for the use of antiVegfs. Studies done in the same area reported a high incidence of RVO [8]. A similar pattern was reported in Oluleye’s series where RVO was the commonest indication [9]. Hypertension as a strong risk factor was implicated in the two studies [8, 9] ^.^ Shuaib, however, reported a different pattern where RVO was the second most common indication for anti Vegf injections after DME [11]. In our series, DME was the second most common indication.

There was a statistically significant association between the type of disease and age group p= 0.001. Wet age related macular degeneration was seen more in those who were 70 years and above in this study.

Hypertension was the commonest systemic condition observed in this study followed by diabetes, while cataract was the commonest ocular comorbidity. All the 61 (31.11%) cases who had short term rise in intraocular pressures received bevacizumab injection. Floaters was the commonest complaint recorded in this series. There was no case of endophthalmitis in this small series; we believe that the strict aseptic conditions as well as 10% Povidone iodine skin prep and 5% drops instillation before and after the injection was effective and contributory.

The supero temporal quadrant was the most preferred site for administering the injections. This is most likely because of the ease of access at this position.

## CONCLUSION

Anti- VEGFs have become an invaluable tool in the management of a number of retinal diseases in our center. However, the cost implications are a hindrance to an increased uptake of this form of treatment. Cheaper alternative preparations should be made available to encourage the uptake. Government in developing countries should be encouraged to bear the health burden of the old age pensioners (OAP). The National Health Insurance which is still in its infancy could assist such therapies.

## Figures and Tables

**Fig. (2):**
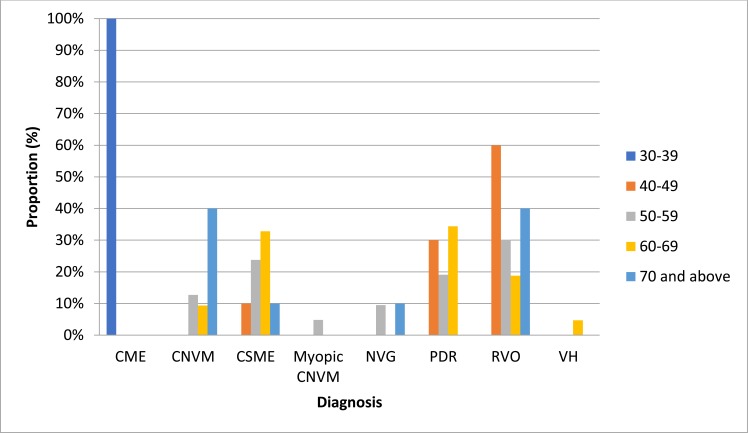
A Bar Chart showing the comparison of Diagnosis and the different Age groups.

**Table 1:** Clinical and Demographics of patients.

**Characteristics**	**Frequency** **n=190**	**Percentage (%)**
**Age group**		
30-39	3	1.58
40-49	30	15.79
50-59	63	33.16
60-69	64	33.68
7 0 and above	30	15.79
** *Mean***	59.56 ± 11.66	
**Sex**		
Male	91	47.89
Female	99	52.11
**TOTAL**	**190**	**100**
**Diagnosis**		
Retinal vein occlusion	61	32.11
Diabetic macular edema	43	22.63
Proliferative diabetic retinopathy	42	22.11
Neovascular age related macular degeneration	26	13.68
Neovascular glaucoma	9	4.74
Myopic choroidal neovascularization	3	1.58
Cystoid macular edema	3	1.58
Vitreous hemorrhage	3	1.58
**TOTAL**	**190**	**100**
**Intraocular Pressure**		
Normal	129	67.89
Raised	61	32.11
** COMPFLAINTS**		
Floaters	40	21.05
Sub conjunctival hemorrhage	6	3.16
Ocular pains	24	12.63
Nil	120	63.16
** TOTAL**	** 190**	** 100**
**INJECTION SITE**		
Supero temporal quadrant	131	68.95
Infero temporal quadrant	44	23.16
Supero nasal quadrant	15	7.89
**TOTAL**	**190**	**100**

**Table 2:** Frequency of Ocular and systemic comorbidities.

**Characteristics**	**Frequency** **n=190**	**Percentage (%)**
**OCULAR CO MORBIDITY**		
Cataract	45	23.68
Pseudo phakia	22	11.58
VH	13	6.84
Nil	110	57.89
**GLAUCOMA**		
Yes	39	20.53
No	151	79.47
**DIABETES**		
Yes	76	40.00
No	114	60.00
**HYPERTENSION**		
Yes	81	42.63
No	109	57.37
**OTHER SYSTEMIC DISEASES**		
hyperlipidemia	3	100.0
